# DolFin: an innovative digital platform for studying Risso’s dolphins in the Northern Ionian Sea (North-eastern Central Mediterranean)

**DOI:** 10.1038/s41598-018-35492-3

**Published:** 2018-11-21

**Authors:** Rosalia Maglietta, Vito Renò, Giulia Cipriano, Carmelo Fanizza, Annalisa Milella, Ettore Stella, Roberto Carlucci

**Affiliations:** 1 Institute of Intelligent Industrial Technologies and Systems for Advanced Manufacturing, National Research Council,Via Amendola 122/D-I, 70126 Bari, Italy; 2 Department of Biology, University of Bari, Via Orabona 4, 70125 Bari, Italy; 3 Jonian Dolphin Conservation, Viale Virgilio 102, 74121 Taranto, Italy

## Abstract

The Risso’s dolphin is a widely distributed species, found in deep temperate and tropical waters. Estimates of its abundance are available in a few regions, details of its distribution are lacking, and its status in the Mediterranean Sea is ranked as Data Deficient by the IUCN Red List. In this paper, a synergy between bio-ecological analysis and innovative strategies has been applied to construct a digital platform, *DolFin*. It contains a collection of sighting data and geo-referred photos of *Grampus griseus*, acquired from 2013 to 2016 in the Gulf of Taranto (Northern Ionian Sea, North-eastern Central Mediterranean Sea), and the first automated tool for *Smart Photo Identification of the Risso’s dolphin* (*SPIR*). This approach provides the capability to collect and analyse significant amounts of data acquired over wide areas and extended periods of time. This effort establishes the baseline for future large-scale studies, essential to providing further information on the distribution of *G. griseus*. Our data and analysis results corroborate the hypothesis of a resident Risso’s dolphin population in the Gulf of Taranto, showing site fidelity in a relatively restricted area characterized by a steep slope to around 800 m in depth, north of the Taranto Valley canyon system.

## Introduction

The Risso’s dolphin *Grampus griseus* (Cuvier, 1812)^1^ is a small Odontocetes distributed from the tropics throughout the temperate regions in both hemispheres^1–5^, living in the deep waters of the continental slope and outer shelf, especially those areas with a steep bottom topography^6–11^. Despite it also being considered a regular inhabitant of the Mediterranean Sea^12–14^, the Risso’s dolphin remains one of the least-known cetacean species in the basin, with its status is ranked as Data Deficient by the IUCN Red List^15^.

To date, the knowledge regarding the presence of *G. griseus* and its distribution has been mainly centred around the Western Mediterranean Sea^7,9,16,17^ and the easternmost part of the Mediterranean basin, as well as in the Greek and Turkish Aegean and Cypriot waters^18–27^, whereas it is lacking in large areas of the Central-eastern regions^10^. As a matter of fact, information on the presence of *G. griseus* in the North-eastern Central Mediterranean Sea is scarce and limited to just one recorded sighting^28^ and stranding data retrieved from the MEDACES database (http://medaces.uv.es/) and the Italian Stranding Network (http://mammiferimarini.unipv.it/)^10^. On the other hand, the need for a more comprehensive understanding of spatial distribution, abundance, site fidelity and habitat use of *G. griseus*, as well as other cetacean species observed in the study area (common bottlenose dolphin *Tursiops truncatus*, striped dolphin *Stenella coeruleoalba*, fin whale *Balaenoptera physalus* and sperm whale *Physeter macrocephalus*)^29^, is especially urgent, considering the requirements under the Habitats Directive, the EU Marine Strategy Framework Directive (MSFD) (https://eur-lex.europa.eu/legal-content/EN/TXT/?qid=1495097018132&uri=CELEX:32017L0845), and the Maritime Spatial Planning Directive (MSPD) (https://eur-lex.europa.eu/legal-content/EN/TXT/?uri=celex%3A32014L0089). In particular, the last two EU Directives clearly indicate an effective development of management strategies, including the assessment of auto-ecology of dolphin and whale species, as well as the evaluation of the anthropic disturbances in their habitats^30,31^.

In this paper, we present a novel investigation, which aims to improve the knowledge of the Risso’s dolphin in the Gulf of Taranto (Northern Ionian Sea, North-eastern Central Mediterranean Sea), by defining its spatial distribution and site fidelity, based on a synergy between bio-ecological information on the species and innovative technological strategies.

Because Risso’s dolphins exhibit long-lasting identifiable natural marks, and, in particular, patterns of scarring and variations in dorsal fin shape, photo-identification (photo-ID) techniques can be used to study its distribution range, site fidelity, association patterns and social structure, as well as abundance and habitat use^32–36^. Photo-ID is a non-invasive technique based on the general hypothesis that each individual is unique within its population, showing several specific physical characteristics useful for its identification. In particular, the number of white scars in the Risso’s dolphins generally increases with age, and older individuals can show a notably white head due to this phenomenon. Several existing catalogues containing dolphin photos taken around the world are, in fact, available (i.e. Dolphin Dock, http://www.dolphindock.com.au/; Morigenos Slovenian Marine Mammal Society, http://www.morigenos.org/en/morigenos/; Associaciò Cetàcea, http://www.associaciocetacea.org/). Unfortunately however, they include little information about Risso’s dolphin sightings.

Indeed, a managed exploitation of innovative technologies could have a positive impact on the studies conducted on poorly known cetaceans. However, the procedures currently applied by domain experts for studies on whales and dolphins are still manual or semi-automated, and deeply bound to the user’s experience. The existing computer-assisted tools for photo-ID require a huge effort, as data needs to be manually imported and pre-processed before being analysed, with high computational cost and low performance accuracy^37–40^. In this regard, DARWIN^37^,which could be considered the state of the art technique among photo-ID algorithms, is based on a semi-automated process to create an approximation of the fin outline of a new dolphin individual. With the cursor, the researcher must trace a general outline of the leading and trailing edges of the dorsal fin, after which the fin outline is repositioned using an active contour. Finally, the fin outline is compared within a database of previously identified dolphin fins. The program presents the researcher with a ranked list of possible matches for comparison with the new fin image, providing also confidence limits for the nearest match to assist with the ID decision. DARWIN works by processing one image at a time portraying a single fin.

This study provides an innovative digital platform, called “DolFin”, containing a catalogue of geo-referred photos and sighting data of *G. griseus*, acquired by our research team in the Gulf of Taranto from 2013 to 2016. In order to build the catalogue, the photo-ID of dolphins was manually performed. Individuals were identified from photos by domain experts through scars and patterns of dolphin fin. Within the catalogue, a subset of dolphins displays photos of both fin sides, facilitating the matching process by researchers viewing nicks on the dorsal fin outline. The catalogue, based on a modern non-relational database (NoSQL), guarantees a perfect integration with other data types and catalogues. An additional contribution of DolFin consists of a fully automated tool for the *Smart Photo Identification of the Risso’s dolphin* (*SPIR*), based on the exploitation of distinctive features of Risso’s dolphins extracted by Speeded Up Robust Features (SURF)^41^. SPIR requires no user interaction and can process multiple images in a single run of the system, thus overcoming the constraints of manual and semi-automated approaches (e.g. DARWIN). In fact, SPIR independently processes either side of the dolphin fin, and it is invariant to the rotation or the scale of the fin in the image^41^. The performance of the SPIR tool was evaluated on test images with different qualities, and acquired under different environmental conditions.

The DolFin platform is freely accessible through a web interface (http://dolfin.ba.issia.cnr.it) to both expert and non-expert users for further studies.

**Figure 1:**
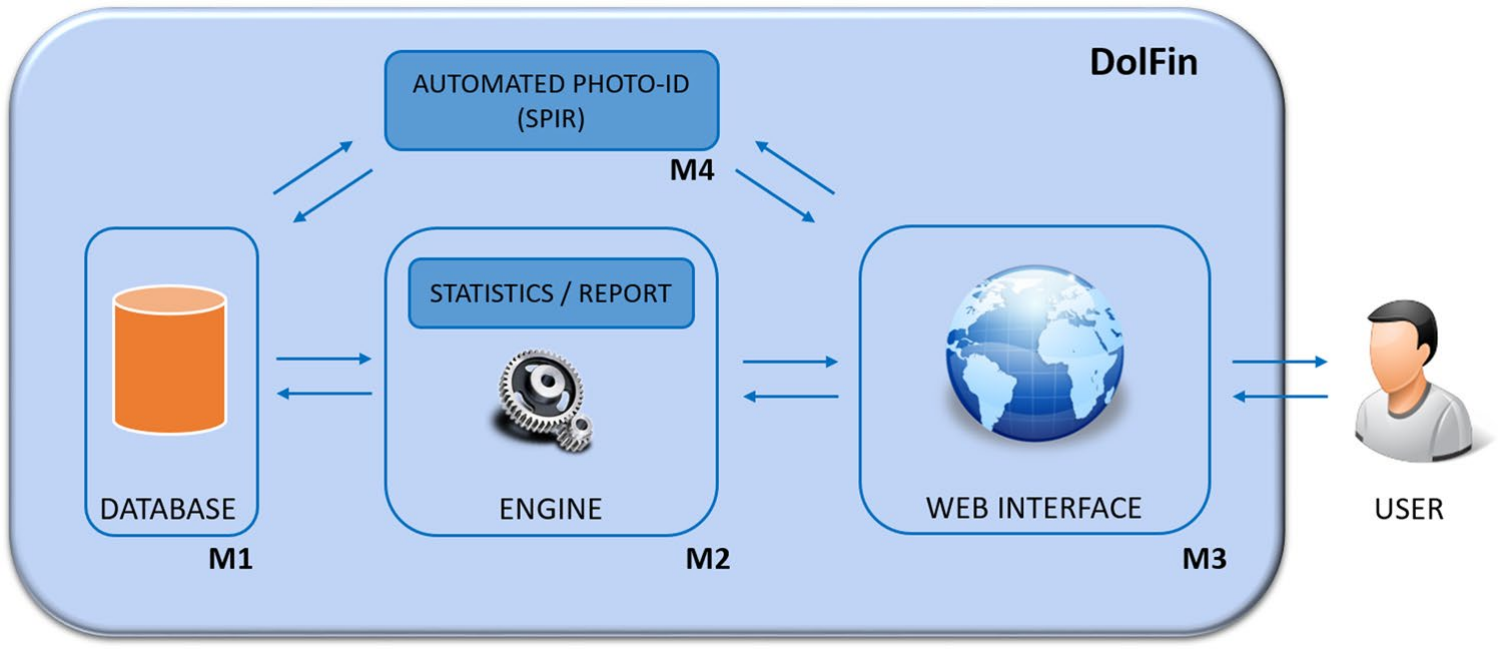
Design of DolFin platform. The engine is responsible for processing requests that come from a web interface accessible by users and manages communication with the database.

A schematic representation of the DolFin platform is shown in Fig. 1 and features four modules: a database, an engine, a web interface and a photo-ID module. The database offers a logical and consistent way to name and organise all available images and information, such as dates and GPS coordinates. The engine is the unit responsible for the analysis of the stored data, in terms of statistics and reports. The automated photo-ID module contains the algorithm for the automated photo identification of new Risso’s dolphin images. Lastly, the web interface offers user-friendly access and data analysis.

## Results

An observational effort lasting around 85 h and covering 595 nautical miles was conducted from 2013 to 2016, providing 17 Risso’s dolphin sightings (3 sightings in 2013, 5 in 2014, 2 in 2015 and 7 in 2016). Sightings mostly occurred in summer (15 of 17) and autumn (2 of 17) whilst none were recorded in winter or spring. A total of 215 *G. griseus* individuals were observed, including 206 adults and juveniles, 7 calves and 2 new-borns. The evidence of calving and nursing females was inferred from calf association without any additional information.

During sightings, the group size varied from 2 to 30 individuals with a mean value of 13 ± 7 dolphins. The depth range varied between 436 and 1000 m with a mean depth of 762 ± 217 m.

Photos of individuals were collected during 11 sightings (2 in 2013, 3 in 2014 and 6 in 2016) in a period of 55 h of observation along 385 nautical miles.

### Statistical analysis of the Risso’s dolphin images in the database

All the Risso’s dolphin images acquired during dedicated surveys in the Gulf of Taranto (Northern Ionian Sea, North-eastern Central Mediterranean Sea) have been manually analysed, cropped and labelled in order to obtain a ground truth. The number of cropped fin images kept in the database is 771, taken from 60 different individuals, each one identified with a given name (see Supplementary Table S1).

**Figure 2:**
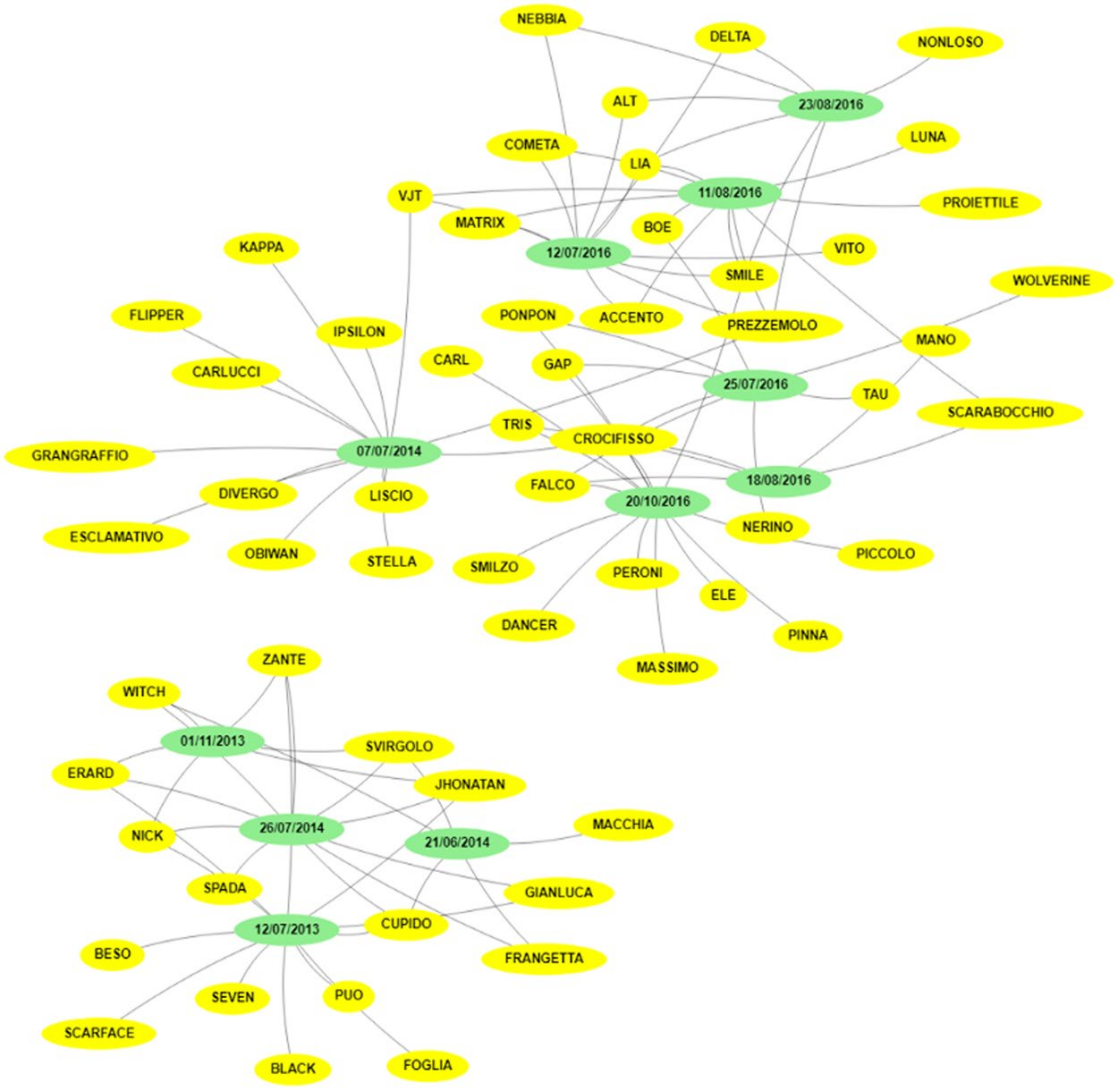
Undirected Graph of Risso’s dolphins sightings and observation dates. The graph shows the connections between the 60 identified dolphins (yellow nodes) and the 11 observation dates (green nodes). The connections are pictured as bidirectional edges.

Figure 2, generated by DolFin, shows the undirected graph of the 60 dolphins (yellow nodes) and the observation dates (green nodes), i.e. the set of connected dolphins and dates, wherein all the edges are bidirectional. The graph is divided into two complementary sub-graphs, meaning that the dolphins observed on the dates in one sub-graph are different from the dolphins seen on the dates in the second sub-graph. Meaningful information on each dolphin is easily accessible using said graph, i.e. when a dolphin is seen, and with which partner(s) during the sighting. Further DolFin outputs (map, recap, pie, word tree), reporting interesting information, can be easily accessed from the web interface.

A new descriptor, called “sighting frequency” of the Risso’s dolphin, is now being introduced to characterise fidelity of the species in waters shallower and deeper than 800 m. Its value is then calculated as the number of sightings of each identified dolphin in a given stratum, normalised over the total number of sightings in the stratum. The analysis is limited to those individuals whose number of sightings is greater than 1 (see Supplementary Table S1, bold *G. griseus* name): 9 dolphins in the bathymetry < 800 m and 17 individuals in the bathymetry > 800 m. The central rank of the sighting frequency was found to be significantly greater in the shallower rather than the deeper stratum (p-value = 0.043 computed using the right-tailed Wilcoxon rank sum test, alpha level set to 0.05), indicating a higher fidelity of the Risso’s dolphin for bathymetries shallower than 800 m.

### SPIR photo-ID results

An innovative tool, called *Smart Photo Identification of the Risso’s dolphin* (*SPIR*), has been used to perform the *G. griseus* photo-ID, based on Speeded Up Robust Features (SURF)^41^ to automatically represent scars and patterns on the dorsal fins.

Among the 60 individuals identified, $d=40\,$dolphins have sufficiently high quality pictures of at least one side of the fin, in terms of resolution, focus and size of the fin (see Supplementary Table S1). Note that among the 20 individuals discarded, 19 were all seen only on one date, with a low number of available photos (e.g. 13 individuals with less than 4 photos; 6 individuals with a number of photos in the range [4,7]; one showing very few scars on the fin and with 18 low quality photos). The total number of *fin models* (i.e. left or right side of the fin) is $m=45$, as images of both fin sides are available for 5 of the $d$ dolphins, whereas, for the other dolphins, only one side was photographed during the surveys. These $d$ dolphins were selected for developing SPIR.

The goal is to assign an identity to the dolphins photographed in new images (test images). To avoid overestimation of SPIR performances, each has been evaluated on test images acquired on dates different from the dates of fin model images. Among the $d$ dolphins, 26 were seen at least twice, meaning that images of these 26 dolphins are available for testing SPIR performance. However, when SPIR is queried with a new dolphin image, it will return its identity choosing from the $d\,$dolphins; in fact the algorithm is designed to work on test images of all the $d\,$dolphins. Moreover, test images having width or height lower than 200 pixels, and with less than 5 SURF features inside the dorsal fin, are not taken into consideration. Lastly 21 out of the 26 dolphins on which to test the algorithm remain, with a total number of 228 fin images (see Supplementary Table S1).

In order to evaluate SPIR performances and to compute its accuracy, the 228 test images are submitted to SPIR. SURF features are computed on each image and matched with those computed on the $m$ fin models. For each test image, the algorithm provides the identity of the best matching dolphin among the $d$ individuals. Lastly, SPIR’s accuracy is 79%, defined as the percentage of correct matches obtained on the reliable test images.

**Figure 3:**
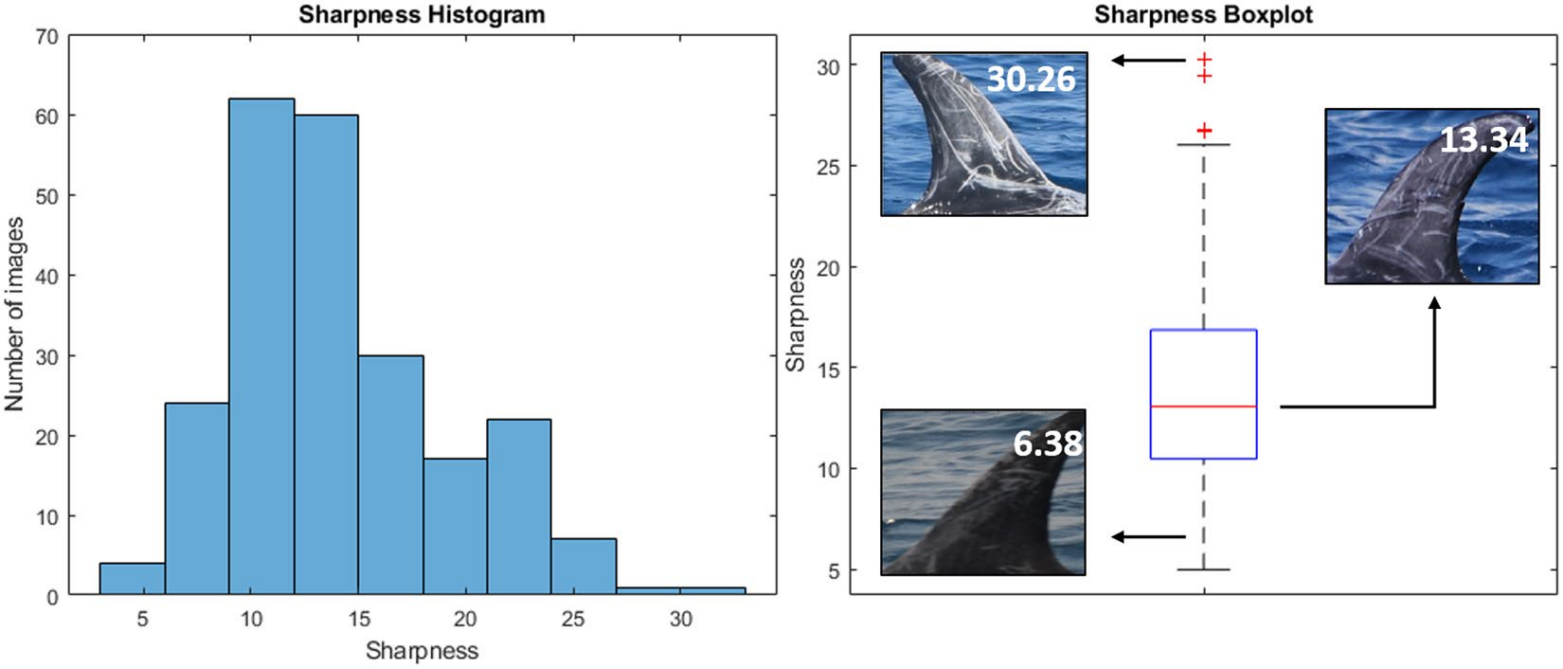
Histogram and boxplot of images sharpness. The sharpness values have been computed on the test set, along with three examples of fin images corresponding to the first quartile, median and fourth quartile. The images do not belong to the same individual and are only representative of the image’s perceived quality with respect to the sharpness value.

The next aim is to highlight how SPIR performances are influenced by the image sharpness (see Methods section) computed over the 228 test images, which varies from 4.97 to 21.93. In Fig. 3 the sharpness of the test images is shown, along with three examples of fin images corresponding to the first quartile (6.38), median (13.34) and an outlier (30.26) of the sharpness values of the test images. The three fins do not belong to the same individual, but were sampled from the test images according to their sharpness values in order to show how this parameter is related to the perceived image quality. Low values of sharpness correspond to blurred images.

**Figure 4:**
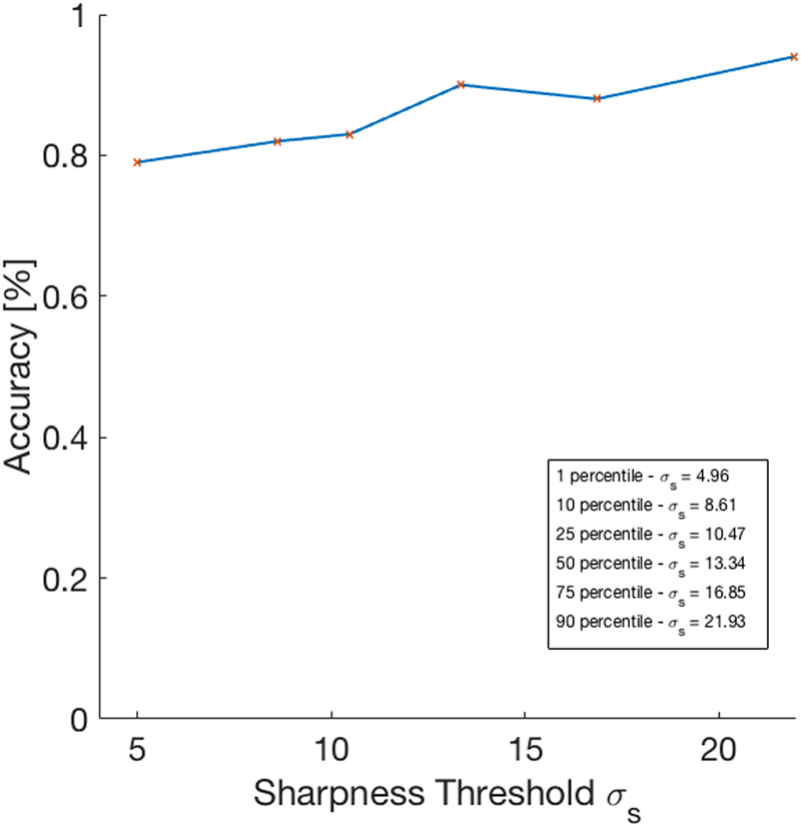
Plot of SPIR’s accuracy vs sharpness threshold ${{\rm{\sigma }}}_{{\rm{s}}}$. The legend shows the values of used thresholds corresponding to the 1, 10, 25, 50, 75, and 90-percentile of the sharpness values computed on the 228 test images.

Figure 4 shows SPIR’s accuracies computed on different subsets of test examples, containing those images, among the 228, having sharpness greater than a threshold ${\sigma }_{s}$. In particular the ${\sigma }_{s}$ used are the 1, 10, 25, 50, 75, and 90-percentile of the sharpness values of the 228 test images. The results show that when ${\sigma }_{s}$ increases (meaning that images with higher sharpness are considered) SPIR’s accuracy increases accordingly, up to 94% evaluated on the best quality images, having sharpness higher than 21.93.

**Figure 5:**
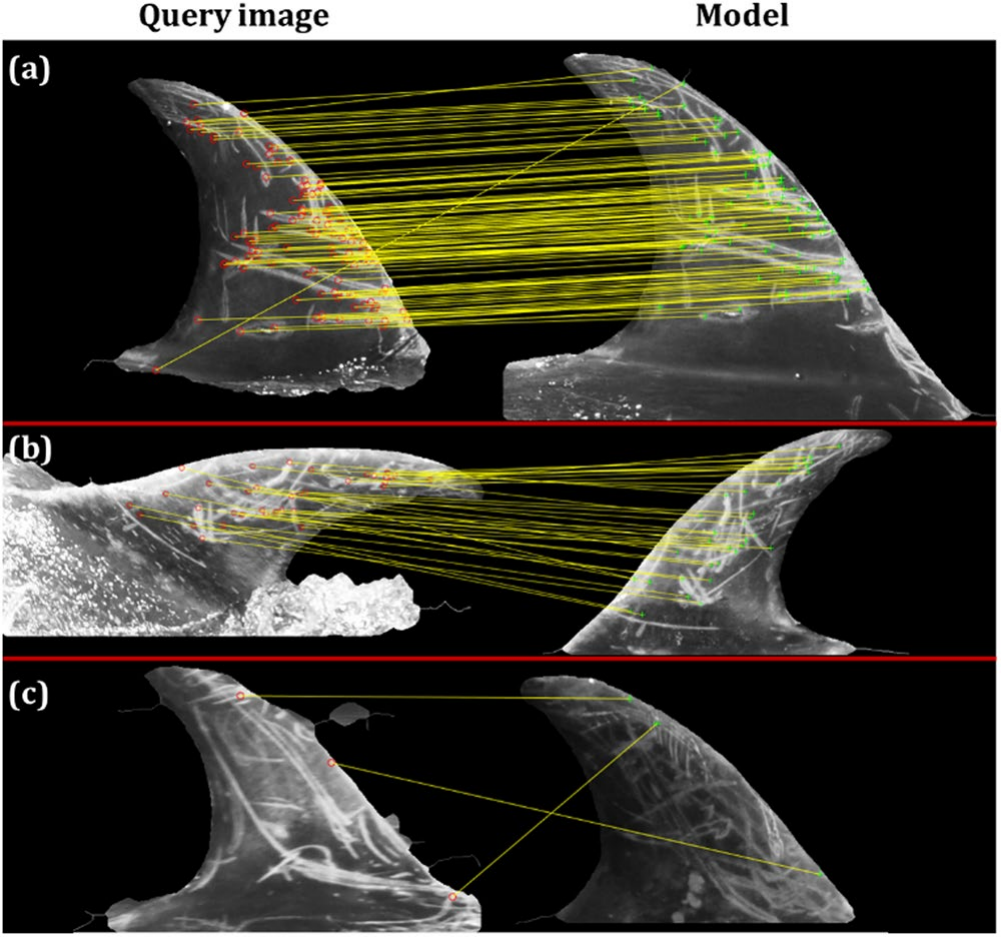
Examples of SPIR output. Three different cases are shown: (**a**) correct prediction, (**b**) correct prediction of rotated input fin and (**c**) incorrect prediction. The new test images are found on the left, while the predicted individuals are shown on the right. Red and green circles represent the exact key-points in which the SURF features were identified.

Figure 5 shows three examples of the qualitative results obtained with SPIR. On the left of the figure the new test images (queries) are shown, while on the right the predicted dolphins are represented. These results have been sampled from the database in order to show different system behaviours: in (a) and (b) the algorithm is able to supply the correct match, while (c) shows an incorrect association. In the first case, two images of similar size and shape are compared, and many SURF features correctly match between the two fins. The second case highlights empirical evidence of the roll (X-plane) and yaw (Z-plane) rotation invariance of SPIR, as well as its capability of filtering additional noise sources (e.g. the water and consequent reflections and/or over-exposed areas. See Methods for details). If one considers the “dorsal fin plane” as the X-Y plane, with Z direction perpendicular to the fin, the query image is rotated along the X and Z-axes, but SPIR correctly matches the query with the corresponding model, without affecting the photo-ID task. Finally, the third image (c) shows an example of wrong prediction due to the fact that there are only three SURF matching features.

## Discussion

The main perspective of this study consists of the combination of computer modelling approaches, statistical models and image-processing algorithms with bio-ecological analysis applied to increase knowledge on the Risso’s dolphin distribution and its habitat use in the investigated area. The result of this effort is the platform DolFin, containing the digital catalogue of Risso’s dolphins sighting data acquired from 2013 to 2016 in the Gulf of Taranto, and the automated photo-ID algorithm SPIR. The platform is easily accessed through a web interface, with no training needed, and it facilitates exchanges among domain scientists. The SPIR algorithm, accessible both on the DolFin platform in interactive mode and as a stand-alone version, enables the user to automatically perform the photo-ID processing of fin images from Risso’s dolphins, reducing the computational time when large amounts of data are analysed.

**Figure 6:**
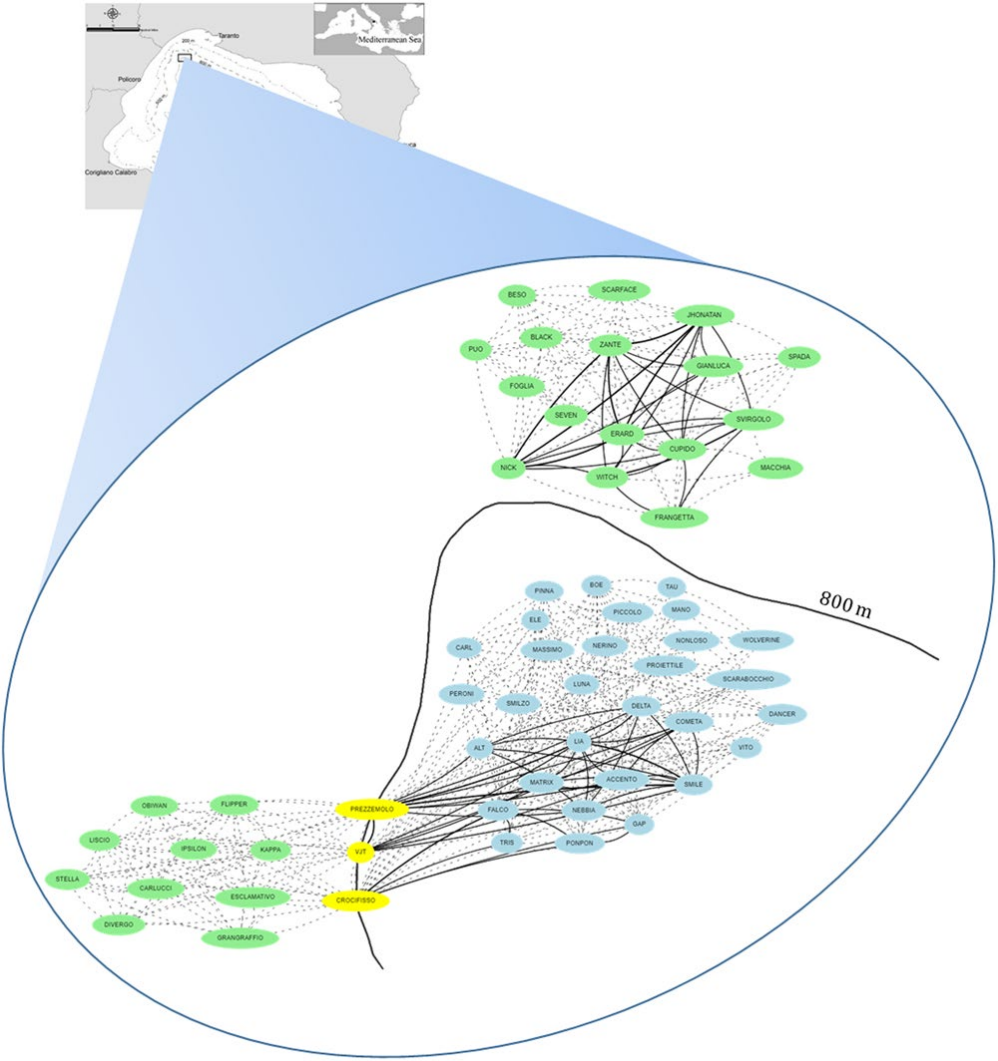
Depth preference of Risso’s dolphins identified in the Northern Ionian Sea (North-eastern Central Mediterranean Sea). In this graph, green nodes represent dolphins seen only in the bathymetry < 800 m, blue nodes refer to those individuals seen only in the bathymetry > 800 m, and yellow nodes represent dolphins seen in both bathymetries. Links between nodes are shown with a dashed line in those cases when two dolphins were only seen on one date, or with a solid line when they were seen on multiple dates.

This study provides unprecedented insights into the spatial distribution of *G. griseus* in the Northern Ionian Sea (North-eastern Central Mediterranean Sea). A total number of 60 Risso’s dolphins has been identified and catalogued within the DolFin database. Although preliminary, this result corroborates the hypothesis of a local population, which, during summer and autumn, resides in a relatively restricted area, characterized by a steep slope of about 800 m in depth in the northernmost part of the Taranto Valley canyon system (Gulf of Taranto) (Fig. 6). However, the identification of a resident population in a relatively small area is an unusual circumstance, and extensive field work over a period of several years is needed to fully address the species’ habitat use^42^. In any case, the observed pattern seems to reflect that reported in the Azores^43^, where females seem to prefer the mesobathyal waters during calving and nursing to reduce the time spent foraging away from the calf, and as well as the risk of shark predation. This pattern closely corresponds to the local *G. griseus* observations recorded during sightings from 2013 to 2016, and the food source availability linked to the geomorphological and hydrographical features of the Gulf of Taranto^44,45^.

These results have very important conservation implications. In fact, despite the presence of *G. griseus* adults, juveniles and calves, no conservation measures to ensure the long-term survival of the species are currently enforced in the area, mostly due to shortcomings in the basic scientific information^28^ available. The Risso’s dolphins in the Northern Ionian Sea could be exposed to elevated levels of anthropogenic threats such as strikes from merchant traffic, disturbance from high intensity military sonar, and exposure to chemical pollution from the nearby harbour of Taranto^30,46,47^, compromising its conservation status and habitat use. In this regard, the main EU environmental policies dealing with the loss of biodiversity indicate the need for a more robust and comprehensive knowledge base that supports policies and actions, which could result in more effective management. In particular, the Marine Strategy Directive Framework requires a biodiversity assessment of species and habitats at ecologically relevant scales, in order to determine whether pressure/state changes fall within safe biological limits^48^. Moreover, within the Marine Strategy Directive Framework, the definitions of indicators and reference points designed to show whether the “Good Environmental Status” (GES) can be achieved in EU waters by 2020^49^, are required.

The DolFin platform seems to be a very promising analytical tool for understanding the distribution and habitat use of *G. griseus* in the study area. It is worth noting that an extensive integration of this catalogue will be essential. In fact, a larger data set of images from both fin sides of new individuals, sampled during additional effort days, will be necessary to evaluate the feasibility of its use in other areas, as well as to enrich data in the study area. Moreover, when applied to sighting data and photo documentation from other areas, it could provide a base of knowledge about species distribution and habitat use, spatially enlarged throughout the Mediterranean Sea and on a global scale.

A future aim will be to integrate other species within the DolFin digital platform, by designing and developing additional innovative methods and technologies for their automated identification.

## Methods

### Study Area

**Figure 7:**
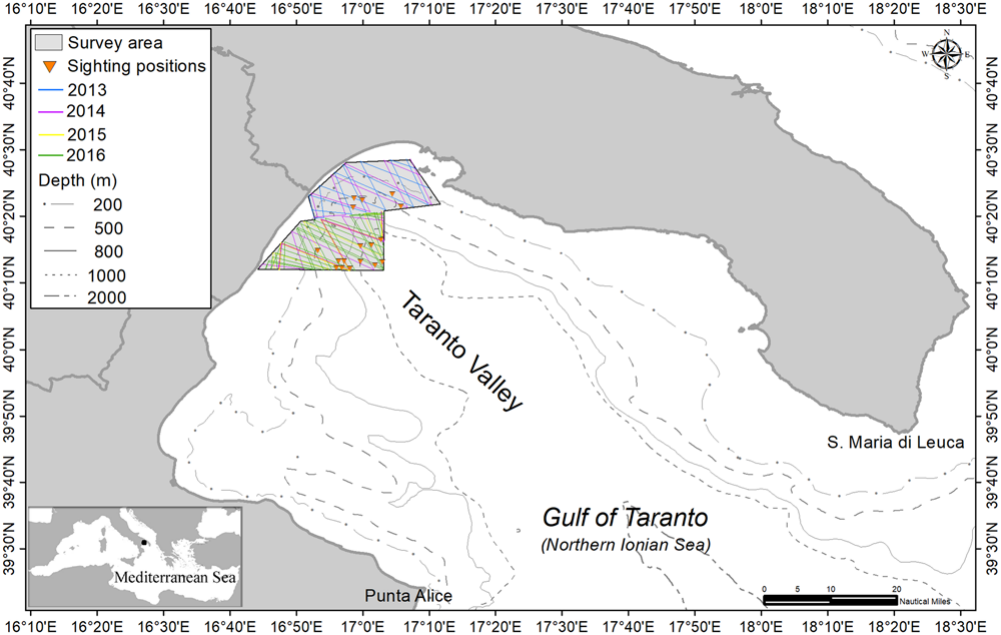
Map of the Gulf of Taranto (Northern Ionian Sea, North-eastern Central Mediterranean Sea). The survey area covered from 2013 to 2016 is shown, with both sighting positions recorded and specific line transects (different colours for different years).

The Gulf of Taranto in the Northern Ionian Sea (North-eastern Central Mediterranean Sea) covers an area of approximately 14,000 km^2^ from Santa Maria di Leuca to Punta Alice, and has a very complex topography (see Fig. 7). A narrow continental shelf with a steep slope and several channels characterise the western sector, while the eastern sector shows descending terraces toward the “Taranto Valley”, a NW-SE submarine canyon with no clear bathymetric connection to a major river system^44,50–52^. This singular morphology involves a complex distribution of water masses with a mixture of both surface and dense bottom waters, as well as the occurrence of high seasonal variability in upwelling currents^53–55^.

### Sightings data

Sightings data for the Risso’s dolphin were collected from 2013 to 2016 during standardized vessel-based surveys carried out using a 12 m catamaran. The sampling effort was approximately 5 h/day along 35 nautical miles (nm). Speed was maintained between 7 and 8 knots and trips only occurred in favourable weather conditions (Douglas scale ≤ 3 and Beaufort scale ≤ 4). A line transect sampling approach was adopted according to Buckland *et al*.^56^ investigating a survey area of about 640 km^2^. Using the Distance 6.0 software, the random and equally spaced zigzag transects were generated daily with an angle of 45 degrees to the x-axis^57^. This proved to be more efficient in terms of reducing effective costs and minimizing off-effort navigation time than the conventional parallel line transects^58^. Off-effort time was generally due to the navigation from the harbours of Taranto or Policoro to the starting point of each random transect line.

The observational team on board consisted of at least three crewmembers rotating roles every 90 minutes. One team member searched for targets within a 180° range, counting the dolphins during each sighting, while the others supported the activities of the former team member, searching in their respective sectors from the track-line to 90° on both the starboard and port sides. Observations were made with either the naked eye or with 7 × 50 binoculars. Once a target was sighted, and if necessary, binoculars were used to identify the species, and video-photo records were made focusing on body markers. All images were taken using a Nikon D3300 camera with a Nikon AF-P Nikkor 70–300 mm, f4,5–6,3 G ED lens. The date, sea weather conditions, depth (m), time of first contact and group size (number of specimens) were also recorded during sightings. To avoid potential interference in dolphin behaviour caused by the presence of the vessel, sampling was interrupted by changing direction when specimens were observed at less than around 50 m^59^. Moreover, all observers maintained a safe distance of not less than 5 m, while lowering speed or interrupting navigation to prevent collisions or possible injuries^60^.

### DolFin platform

The proposed database does not follow a strict relational model and is particularly suited for dealing with a large amount of data that evolves over time. Our data model comprises the following attributes:

1. Dolphin Name – the name given to the individual;
2. Input Image – the path (local or remote) of the full processed resolution image;
3. Cropped Fin – the path (local or remote) of the dorsal fin image;
4. Observation date – the date when the sighting occurred;
5. GPS – the GPS coordinates associated with the image;
6. Codename – a flag indicating the acquisition campaign of the images.

These represent the minimal attributes necessary to extract meaningful statistics and reports (using M2), and to perform automated photo identification (using M4) (see Fig. 1).

The module Engine M2 implements the following functions:

1. Statistics showing relations between the images of each individual and observation date: number of images for each dolphin available per date (see Recap of M3);
2. Statistics showing how many images are provided per individual: number of images available for each individual (see Pie of M3);
3. Statistics showing the relations between individuals and observation dates: a) number of different dates on which a dolphin was observed; b) number of dolphins observed on a specific date (see Graph + Scatter and Word Tree of M3).

The access to these results is available through the module Web Interface M3, given to the user upon first connecting, and consisting of:

1. DB Viewer – a database viewer that allows the user to surf the catalogue.
2. Map – a graphical recap of GPS data related to the observations.
3. Recap – a table showing the number of images of each dolphin for each observation date (see item 1 of M2).
4. Pie – a pie chart showing how many images are provided of each individual (see item 2 of M2).
5. Graph + Scatter – both a scatter chart and a graph highlighting which dolphins are observed on each date and all the dates in which a dolphin is observed (see item 3 of M2).
6. Word Trees – another data representation that immediately shows the most viewed individuals over time (see item 3 of M2): showing a word cloud that depicts the name of each dolphin with a different font size relating to the number of sightings.

Finally, the Automated Photo-ID (SPIR) M4 is presented as an interactive web page to analyse single images of Risso’s dolphins in real time.

### Automated Photo Identification Tool

**Figure 8:**
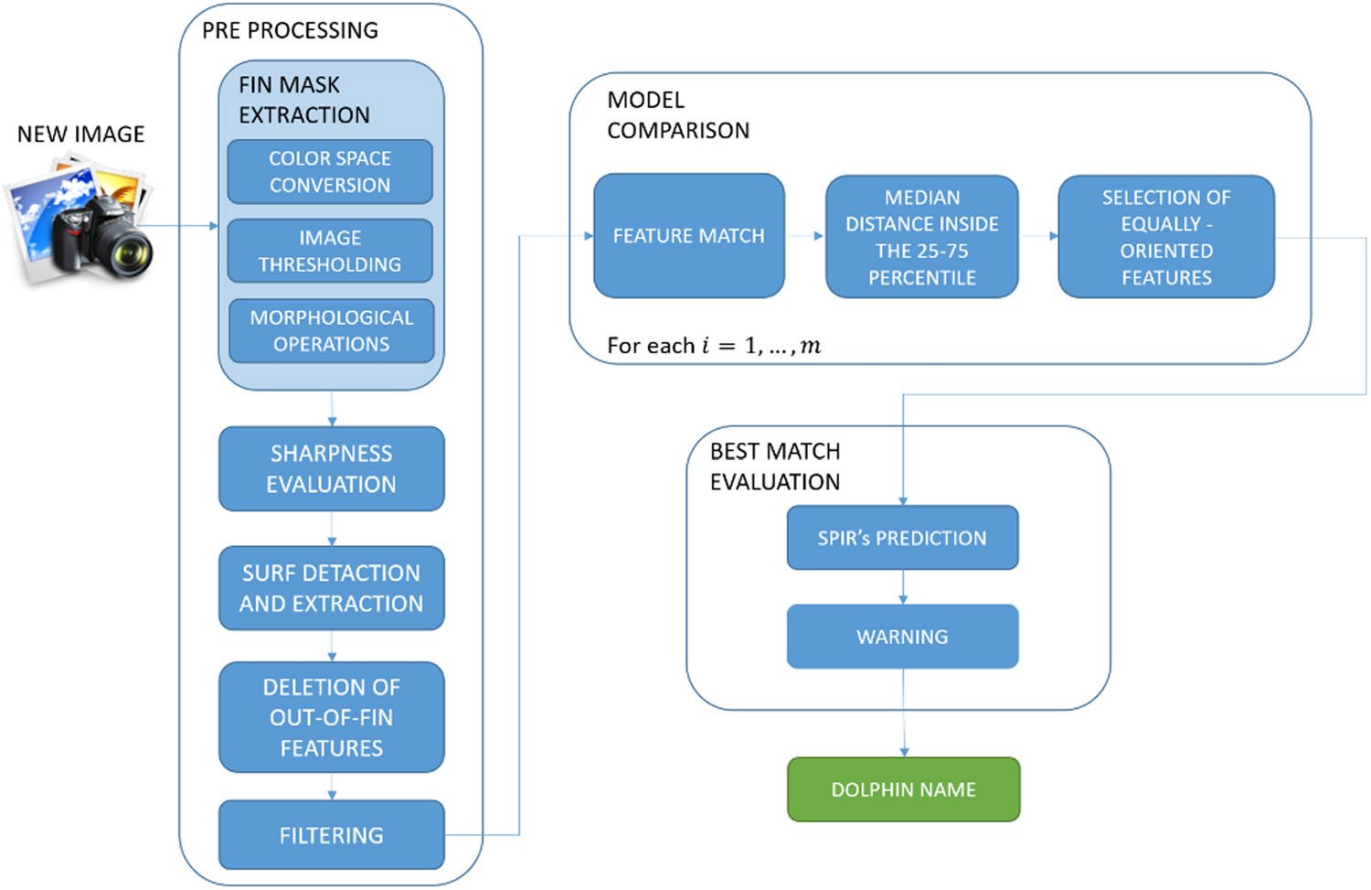
Block diagram of the SPIR algorithm.

A diagram of SPIR is graphically summarized in Fig. 8 and details of each building block are reported in the following paragraph. In the first step of the procedure, a new image ${I}_{new}$ is appropriately pre-processed, and the fin mask is extracted using the following three steps:

1. *Colour space conversion* from RGB to CIE-L*a*b*, that transforms the colour information into a mathematical description of human visual perception through the representation of Lightness (L*), green-red (a*) and blue-yellow (b*) colour opponents;
2. *Image thresholding* with the Otsu technique, which automatically establishes the optimal cut-off value minimizing the intra-class variance between two classes, background and foreground. This approach is justified by the fact that a* and b* coordinates are able to clearly divide dorsal fin pixels from those belonging to the sea;
3. *Morphological operations*, opening and closing, used for noise removal purposes.

*Sharpness evaluation* is performed to quantitatively estimate the sharpness of the image is (or, equivalently, the amount of blur inside the fin) in order to discard heavily corrupted images, whose subsequent SURF computation would translate into unusable results with a high level of confidence. This task is performed by computing the standard deviation of the Laplacian-kernel convolved image, and returning a real number that estimates its sharpness.

*SURF detection and extraction* follow. This procedure detects and describes maximally informative points and their surroundings, regardless of the scale and rotation of the image, ensuring a high repeatability of the task. Moreover, its computation is extremely fast as compared with other state-of-the-art approaches, thus making it a reasonable choice in the development of more complex algorithms^61–63^. *Deletion of out-of-fin features* is required to delete features falling outside the fin.

A *filtering* step is then implemented to avoid inconsistencies and unreliable responses of the subsequent software modules: only dorsal fin images with at least a $200$ pixels width, a $200$ pixels height and $5$ SURF features are allowed to pass through model evaluation.

Once an image passes the pre-processing steps, it is enriched with its SURF description and represented as a pair $({I}_{new},{S}_{new})$.

The algorithm has access to the set $M$ of $m$ images, representing the $m$ fin models of known dolphins, pre-processed and enriched with their SURF feature descriptions, following the same algorithm used for ${I}_{new}$:

$$M=\{({I}_{1},{S}_{1})\,,({I}_{2},{S}_{2}),\ldots ,({I}_{m},{S}_{m})\}.$$

In order to predict the identity of the new dolphin, a *model comparison* step is required, comparing $({I}_{new},{S}_{new})$ with each image $({I}_{i},{S}_{i}),\,i=1,\ldots ,m$. The following operations are performed to make the comparison:

1. An operation of *feature match* between ${S}_{new}$ and ${S}_{i}$, for$\,i=1,\ldots ,m$, takes place to identify the ${k}_{i}\,$matching points between the two images, if existing^41^. The coordinates of the pair of matched points, their relative distance (computed by L2-norm) and orientation are respectively stored in the arrays $\,{P}_{i}$, ${D}_{i}$ and ${O}_{i}$, each of them containing ${k}_{i}\,$elements. The *median distance* ${d}_{i}^{\ast }\,$*inside the 25*^*th*^ *–75*^*th*^ *percentile* interval of$\,{D}_{i}$ is computed.
2. The second step consists of the *selection of equally oriented features* among the ${k}_{i}$ matching points. For$\,j=1,\ldots ,{k}_{i}$, let us select $\,{o}_{j}=({o}_{j}^{(new)},\,{o}_{j}^{(i)})\in {O}_{i}$, containing the orientations of the matched points ${p}_{j}\in {P}_{i}\,$and the following quantity is therefore computed:

$${\omega }_{j}=round({o}_{j}^{(new)}-{o}_{j}^{(i)})$$

Only the$\,{k}_{i}^{\ast }$ features having ${\omega }_{j}=0$ are kept in the array$\,{P}_{i}$* while the others are, then, discarded.

This final step of the pipeline, consisting of a *best match evaluation*, is responsible for associating a label to the unknown fin image, thus completing the photo identification process. The *SPIR’s prediction* is the fin model ${i}^{s}$ with the highest number ${k}_{i}^{\ast }\,$of equally-oriented matched features with the new image, that is:

$${i}^{s}=\mathop{{\rm{argmax}}}\limits_{i=1,\ldots ,m}(cardinality({P}_{i}^{\ast }))$$

If $cardinality\,({P}_{i}^{\ast })\le 4$, SPIR warns the user about the reliability of the prediction. If two or more models have the same maximum value of $P{i}^{\ast }$ cardinality, the model with the minimum median distance $d{i}^{\ast }$ is selected. If the minimum median distances are equal, SPIR provides a *warning* to the user.

## Electronic supplementary material

Supplementary Materials

## Data Availability

The datasets generated during and/or analysed for the current study are available from the corresponding author upon request. A stand-alone version of SPIR, developed using MATLAB software (MathWorks, Natick, MA), is also available upon request.
