# Supplementary material for: DolFin: an innovative digital platform for studying Risso’s dolphins in the Northern Ionian Sea (North-eastern Central Mediterranean)
Source: Sci Rep. 2018 Nov 21;8:17185. doi: 10.1038/s41598-018-35492-3 (PMC6249232; doi:10.1038/s41598-018-35492-3)
Supplement: Supplementary file 1 — Supplementary Materials [file 41598_2018_35492_MOESM1_ESM.pdf]

DolFin: an innovative digital platform for studying Risso's dolphins in the Northern Ionian Sea (North-eastern Central Mediterranean)

Rosalia Maglietta[1], Vito Renò[1], Giulia Cipriano[2], Carmelo Fanizza[3], Annalisa Milella[1], Ettore Stella[1], Roberto Carlucci[2]*

(1) INSTITUTE OF INTELLIGENT INDUSTRIAL TECHNOLOGIES AND SYSTEMS FOR ADVANCED MANUFACTURING, NATIONAL RESEARCH COUNCIL, VIA AMENDOLA 122/D-I, 70126 BARI, ITALY

(2) DEPARTMENT OF BIOLOGY, UNIVERSITY OF BARI, VIA ORABONA 4, 70125 BARI, ITALY

(3) JONIAN DOLPHIN CONSERVATION, VIALE VIRGILIO 102, 74121 TARANTO, ITALY

* corresponding author. e-mail to: rosalia.maglietta@cnr.it

Supplementary Table S1. Details on the sightings of the 60 *G. griseus* dolphins stored in the DolFin database. Each dolphin is identified with a given name, number of images available, observation dates, GPS data, number of sightings and depth. Bold *G. griseus* name are used for highlighting dolphins with multiple sightings (greater than 1), which are involved in the bathymetry analysis (see Results section, page 5). Finally, the last column, entitled Model, shows if there is any image available as SPIR's model (in total m = 45) for an individual (L means left side of the fin, R means right side of the fin, * means that no model is available for the dolphin). Red colour highlights the 21 dolphins captured in the 228 test set images.

G. griseus name	# images	observation dates	GPS data	# of sightings	depth (m)	Model
**ACCENTO**	7	12/07/2016	(40.215 N - 17.013017 E)	2	1000	L
		11/08/2016	(40.188883 N - 16.98405 E)		945	
**ALT**	18	12/07/2016	(40.215 N - 17.013017 E)	2	1000	R
		23/08/2016	(40.199717 N - 17.07135 E)		1000	
BESO	7	12/07/2013	(40.378333 N - 16.978817 E)	1	500	*
BLACK	18	12/07/2013	(40.378333 N - 16.978817 E)	1	500	*
**BOE**	3	25/07/2016	(40.175383 N - 17.0023 E)	2	1000	*
		11/08/2016	(40.188883 N - 16.98405 E)		945	
CARL	1	20/10/2016	(40.21455 N - 17.058017 E)	1	1000	L
CARLUCCI	8	07/07/2014	(40.203667 N - 16.947533 E)	1	782	R
**COMETA**	6	12/07/2016	(40.215 N - 17.013017 E)	2	1000	R
		11/08/2016	(40.188883 N - 16.98405 E)		945	
**CROCIFISSO**	11	07/07/2014	(40.203667 N - 16.947533 E)	4	782	L
		25/07/2016	(40.175383 N - 17.0023 E)		1000	
		18/08/2016	(40.178483 N - 17.0342 E)		1000	
		20/10/2016	(40.21455 N - 17.058017 E)		1000	
**CUPIDO**	96	12/07/2013	(40.378333 N - 16.978817 E)	3	500	R
		21/06/2014	(40.355033 N - 16.975933 E)		559	
		26/07/2014	(40.388167 N - 17.0744 E)		436	
DANCER	3	20/10/2016	(40.21455 N - 17.058017 E)	1	1000	R
**DELTA**	14	12/07/2016	(40.215 N - 17.013017 E)	2	1000	L

		23/08/2016	(40.199717 N - 17.07135 E)		1000	
DIVERGO	7	07/07/2014	(40.203667 N - 16.947533 E)	1	782	*
ELE	1	20/10/2016	(40.21455 N - 17.058017 E)	1	1000	*
**ERARD**	88	12/07/2013	(40.378333 N - 16.978817 E)	3	500	R
		01/11/2013	(40.3575 N - 17.096433 E)		475	
		26/07/2014	(40.388167 N - 17.0744 E)		436	
ESCLAMATIVO	1	07/07/2014	(40.203667 N - 16.947533 E)	1	782	*
**FALCO**	5	25/07/2016	(40.175383 N - 17.0023 E)	3	1000	L
		18/08/2016	(40.178483 N - 17.0342 E)		1000	
		20/10/2016	(40.21455 N - 17.058017 E)		1000	
FLIPPER	1	07/07/2014	(40.203667 N - 16.947533 E)	1	782	*
FOGLIA	6	12/07/2013	(40.378333 N - 16.978817 E)	1	500	*
**FRANGETTA**	51	21/06/2014	(40.355033 N - 16.975933 E)	2	559	L
		26/07/2014	(40.388167 N - 17.0744 E)		436	
**GAP**	18	25/07/2016	(40.175383 N - 17.0023 E)	2	1000	L/R
		20/10/2016	(40.21455 N - 17.058017 E)		1000	
**GIANLUCA**	47	12/07/2013	(40.378333 N - 16.978817 E)	2	500	L/R
		26/07/2014	(40.388167 N - 17.0744 E)		436	
GRANGRAFFIO	2	07/07/2014	(40.203667 N - 16.947533 E)	1	782	*
IPSILON	11	07/07/2014	(40.203667 N - 16.947533 E)	1	782	R
**JHONATAN**	14	12/07/2013	(40.378333 N - 16.978817 E)	3	500	R
		01/11/2013	(40.3575 N - 17.096433 E)		475	
		26/07/2014	(40.388167 N - 17.0744 E)		436	
KAPPA	10	07/07/2014	(40.203667 N - 16.947533 E)	1	782	L/R
**LIA**	5	12/07/2016	(40.215 N - 17.013017 E)	3	1000	L
		11/08/2016	(40.188883 N - 16.98405 E)		945	
		23/08/2016	(40.199717 N - 17.07135 E)		1000	
LISCIO	5	07/07/2014	(40.203667 N - 16.947533 E)	1	782	*
LUNA	2	11/08/2016	(40.188883 N - 16.98405 E)	1	945	*
MACCHIA	2	21/06/2014	(40.355033 N - 16.975933 E)	1	559	*
MANO	4	18/08/2016	(40.178483 N - 17.0342 E)	1	1000	L
MASSIMO	3	20/10/2016	(40.21455 N - 17.058017 E)	1	1000	L/R
**MATRIX**	6	12/07/2016	(40.215 N - 17.013017 E)	2	1000	L
		11/08/2016	(40.188883 N - 16.98405 E)		945	
**NEBBIA**	6	12/07/2016	(40.215 N - 17.013017 E)	2	1000	R
		23/08/2016	(40.199717 N - 17.07135 E)		1000	
NERINO	4	25/07/2016	(40.175383 N - 17.0023 E)	1	1000	*
**NICK**	37	12/07/2013	(40.378333 N - 16.978817 E)	3	500	R
		01/11/2013	(40.3575 N - 17.096433 E)		475	
		26/07/2014	(40.388167 N - 17.0744 E)		436	
NONLOSO	1	23/08/2016	(40.199717 N - 17.07135 E)	1	1000	*

OBIWAN	5	07/07/2014	(40.203667 N - 16.947533 E)	1	782	L
PERONI	23	20/10/2016	(40.21455 N - 17.058017 E)	1	1000	R
PICCOLO	2	20/10/2016	(40.21455 N - 17.058017 E)	1	1000	*
PINNA	3	20/10/2016	(40.21455 N - 17.058017 E)	1	1000	*
**PONPON**	12	25/07/2016	(40.175383 N - 17.0023 E)	2	1000	R
		20/10/2016	(40.21455 N - 17.058017 E)		1000	
**PREZZEMOLO**	26	07/07/2014	(40.203667 N - 16.947533 E)	4	782	R
		12/07/2016	(40.215 N - 17.013017 E)		1000	
		11/08/2016	(40.188883 N - 16.98405 E)		945	
		23/08/2016	(40.199717 N - 17.07135 E)		1000	
PROIETTILE	2	11/08/2016	(40.188883 N - 16.98405 E)	1	945	R
PUO	5	12/07/2013	(40.378333 N - 16.978817 E)	1	500	*
**SCARABOCCHIO**	3	11/08/2016	(40.188883 N - 16.98405 E)	2	945	L
		18/08/2016	(40.178483 N - 17.0342 E)		1000	
SCARFACE	2	12/07/2013	(40.378333 N - 16.978817 E)	1	500	*
SEVEN	9	12/07/2013	(40.378333 N - 16.978817 E)	1	500	L
**SMILE**	9	12/07/2016	(40.215 N - 17.013017 E)	4	1000	R
		11/08/2016	(40.188883 N - 16.98405 E)		945	
		23/08/2016	(40.199717 N - 17.07135 E)		1000	
		20/10/2016	(40.21455 N - 17.058017 E)		1000	
SMILZO	3	20/10/2016	(40.21455 N - 17.058017 E)	1	1000	L
SPADA	12	26/07/2014	(40.388167 N - 17.0744 E)	1	436	R
STELLA	1	07/07/2014	(40.203667 N - 16.947533 E)	1	782	*
**SVIRGOLO**	26	01/11/2013	(40.3575 N - 17.096433 E)	3	475	L
		21/06/2014	(40.355033 N - 16.975933 E)		559	
		26/07/2014	(40.388167 N - 17.0744 E)		436	
TAU	10	25/07/2016	(40.175383 N - 17.0023 E)	1	1000	R
**TRIS**	6	18/08/2016	(40.178483 N - 17.0342 E)	2	1000	R
		20/10/2016	(40.21455 N - 17.058017 E)		1000	
VITO	1	12/07/2016	(40.215 N - 17.013017 E)	1	1000	*
**VJT**	5	07/07/2014	(40.203667 N - 16.947533 E)	3	782	L
		12/07/2016	(40.215 N - 17.013017 E)		1000	
		11/08/2016	(40.188883 N - 16.98405 E)		945	
**WITCH**	21	01/11/2013	(40.3575 N - 17.096433 E)	3	475	R
		21/06/2014	(40.355033 N - 16.975933 E)		559	
		26/07/2014	(40.388167 N - 17.0744 E)		436	
WOLVERINE	9	25/07/2016	(40.175383 N - 17.0023 E)	1	1000	L
**ZANTE**	47	12/07/2013	(40.378333 N - 16.978817 E)	3	500	L/R
		01/11/2013	(40.3575 N - 17.096433 E)		475	
		26/07/2014	(40.388167 N - 17.0744 E)		436	
TOTAL	771					
